# The Alarming Eastward Front of Cassava Mosaic Disease Reported in Guinea and Sierra Leone Reaches Western Côte d’Ivoire

**DOI:** 10.3390/v18030319

**Published:** 2026-03-04

**Authors:** Justin S. Pita, Fidèle Tiendrébéogo, Angela O. Eni, William J.-L. Amoakon, Bekanvié S. M. Kouakou, Mariam Combala, Aya Ange Nate Yoboue, Guy R. Eboulem, Daniel H. Otron, Maïmouna M. Koné, John Steven S. Seka, Richard A. K. Aka, Merveille Koissi Savi, Cyrielle Ndougonna, Nazaire K. Kouassi

**Affiliations:** 1 Laboratoire d’Innovation pour la Santé des Plantes, UFR Biosciences, Université Félix Houphouët-Boigny, Abidjan 22 BP 582, Côte d’Ivoire; 2 The Central and West African Virus Epidemiology (W.A.V.E.) for Food Security Program, Pôle Scientifique et D’innovation, Université Félix Houphouët-Boigny, Bingerville 22 BP 582, Côte d’Ivoire

**Keywords:** cassava mosaic disease, cassava mosaic begomoviruses, EACMV-Ug, Côte d’Ivoire

## Abstract

Cassava mosaic begomoviruses are a major threat to cassava cultivation in Africa. The virulent Ugandan variant of the East African cassava mosaic virus (EACMV-Ug), which caused substantial damage to cassava production in Uganda in the 1990s and which was previously confined to East and Central Africa, was recently found to be well established in Guinea and Sierra Leone in West Africa. Molecular analysis of cassava leaf samples from a nationwide cassava fields survey conducted in Côte d’Ivoire in 2022 suggested the absence of EACMV-Ug in the country in 2022. Given the proximity of some confirmed EACMV-Ug infected locations in Guinea to Côte d’Ivoire, we conducted another survey in 2025 along the entire western border of Côte d’Ivoire, bordering Guinea and Liberia, to update the status of EACMV-Ug in the country. Molecular analysis of the leaf samples collected confirmed the presence of EACMV-Ug in Côte d’Ivoire for the first time, along with other begomoviruses. The infection rate of EACMV-Ug along the Liberian border was higher (28.85%) than the 17.07% observed along the Guinean border. African cassava mosaic virus (ACMV) and East African cassava mosaic Cameroon virus (EACMCMV) were detected both as a single infection and in double co-infections (ACMV+EACMCMV) in some plants, whereas EACMV-Ug was found as a double co-infection (EACMCMV+EACMV-Ug) and as a triple co-infection (ACMV+EACMCMV+EACMV-Ug). Our results also show that all the cassava varieties grown in the surveyed locations were susceptible to EACMV-Ug. Epidemiological assessment of cassava fields revealed that the incidence and severity of cassava mosaic disease (CMD) were significantly higher along the Liberian border compared to the Guinean border. However, whitefly populations were relatively low across the entire area surveyed. Furthermore, we found that the spread of CMD in the survey area was mainly through the use of infected cassava cuttings for the establishment of new farms. Based on these results, it is imperative to conduct an urgent nationwide cassava fields survey to assess the extent of EACMV-Ug spread in Côte d’Ivoire and implement containment measures to stop further spread.

## 1. Introduction

Cassava (*Manihot esculenta* Crantz), a member of the Euphorbiaceae family, is one of the most important staple crops cultivated in tropical and subtropical regions of the world. Its resilience to poor soils conditions and adaptability to the effects of climate change makes it a crucial food source for developing countries [1]. Beyond its nutritional value, cassava serves as a versatile raw material for the production of various industrial goods, including ethanol, biofuels and bread [2].

In 2023, global cassava production was estimated to be approximately 333 million tons [3]. In Africa, Nigeria leads as the top cassava producer, contributing around 62 million tons [3]. In Côte d’Ivoire, where the crop is predominantly used in the preparation of Attiéké, a traditional dish widely appreciated and consumed daily by Ivorians, cassava ranks as the second most important food crop after yam, with an annual production of about 7.2 million tons [3,4].

Despite its socio-economic importance, cassava cultivation remains constrained by the scarcity of healthy planting material and the continuous pressure exerted by a broad spectrum of pests and pathogens, including insects, green mites, bacteria, fungi, phytoplasmas, and viruses [5]. Among these threats, viral infections are particularly challenging to control and can affect both yield and the quality of the edible roots.

Two viral diseases, cassava mosaic disease (CMD) and cassava brown streak disease (CBSD) threaten cassava production globally. The typical CMD chlorotic mosaic patterns on cassava leaves impair photosynthesis, leading to substantial yield reduction [6]. In contrast, CBSD induces more severe symptoms, which are evident not only on the foliage and stems but also on the tuberous roots. While CMD is widespread across many African countries, CBSD remains geographically restricted to East and Central Africa [7]. In susceptible varieties, CBSD and CMD can cause yield losses of up to 100% and 90% respectively [8,9].

The viruses responsible for CMD belong to the Geminiviridae family, specifically to the Begomovirus genus. Among the 445 Begomovirus species currently known to infect plants, eleven infect cassava, with nine of these species found in Africa “https://ictv.global/report/chapter/geminiviridae/geminiviridae/begomovirus (accessed on 9 December 2025)’’. The two most prevalent and widespread species are *Begomovirus manihotis* (African cassava mosaic virus; ACMV) and *Begomovirus manihotisafricaense* (East African cassava mosaic virus; EACMV). These viruses possess bipartite genomes composed of two single-stranded DNA molecules: DNA-A, which governs viral replication, encapsidation and transmission, and DNA-B, which facilitates the systemic movement of the virus within the host plant [10].

CMD is primarily propagated through the use of infected cassava cuttings as planting material. Additionally, CMD is transmitted by the whitefly *Bemisia tabaci*, the principal insect vector responsible for the spread of the disease [8].

Control strategies implemented to date against these viruses include the use of CMD-resistant cassava varieties, sanitization/multiplication of clean plant material through meristem tip/in vitro culture, and the application of insecticides targeting whitefly populations [11]. Despite these efforts, the emergence of new viral variants continues to threaten cassava production. A prominent example is the Ugandan variant of *Begomovirus manihotisafricaense*, formerly known as the East African cassava mosaic virus-Ugandan variant (EACMV-Ug), which was first reported in Uganda during the 1990s [12]. This highly virulent recombinant triggered a CMD epidemic that severely affected cassava cultivation across Uganda.

In West Africa, EACMV-Ug was first detected in Burkina Faso by Tiendrebéogo et al. [13], but was not established in the country [14]. Sixteen years later, Combala et al. [15] and Saffa et al. [16], reported the presence of the Ugandan variant in several regions of Guinea bordering western Côte d’Ivoire and in Sierra Leone, and raised an alert on an eastward front about CMD developing in West Africa. The western region of Côte d’Ivoire is a key cassava-producing area for the country. Considering the geographic proximity of Guinea to Côte d’Ivoire, the frequent trade between the two countries and the virulent nature of the EACMV-Ug variant, it was imperative to conduct a preemptive cassava field survey to assess the status of EACMV-Ug in the western region of Côte d’Ivoire bordering Guinea and Liberia. This study aimed to generate up-to-date epidemiological data to inform Côte d’Ivoire’s preparedness against the eastward CMD front progressing towards the country.

## 2. Materials and Methods

### 2.1. Countrywide Cassava Field Survey Conducted in Côte d’Ivoire in 2022

In January 2022, a nationwide survey of cassava fields was conducted across Côte d’Ivoire, following the main road network. Cassava fields (3–6 months old) were surveyed at intervals of approximately 10 km, and 30 plants were assessed per field (15 plants along two intersecting diagonals). Symptom severity was evaluated according to the protocol described by Sseruwagi et al. [17] and Eni et al. [18], and based on visual assessment, leaf samples were collected from symptomatic and asymptomatic plants. Molecular analysis of the leaf samples was conducted as detailed in Section 2.4 and Section 2.5.

### 2.2. Cassava Field Survey Conducted in the Western Border of Côte d’Ivoire

Due to EACMV-Ug threats from Guinea, we conducted another cassava field survey in April 2025, targeting the western region of Côte d’Ivoire, along the borders with Guinea and Liberia. The surveyed areas were located in four different agroecological zones, agroecological zone II and III in the southwest bordering Liberia and agroecological zone V and VI in the northwest bordering Guinea. Cassava fields of 3 to 6 months were evaluated at intervals of approximately 5–10 km along the primary and secondary roads. In each field visited, 30 cassava plants were assessed visually, with 15 plants randomly selected along each of two intersecting diagonals.

For each site, geographical coordinates including longitude, latitude and altitude were recorded using a global positioning system (GPS; Garmin OREGON 600). Epidemiological data included the age of the cassava plants, presence or absence of CMD symptoms, severity of CMD symptoms, number of whiteflies on the five topmost leaves of each plant, and mode of CMD transmission (whitefly-borne or cuttings-borne). The agronomic description of the cassava varieties present in each field was collected using the KoboCollect application (version 2024.2.4).

In each field, one to four cassava leaf samples were collected depending on the phytosanitary status of the plants, severity of CMD symptoms, and the number of cassava varieties present in the field. We aimed to collect samples from at least one asymptomatic plant and one from each plant showing very severe, severe and or/mild CMD symptoms. The samples collected were placed in labeled envelopes for subsequent molecular analysis. Whitefly density was determined by counting adult whiteflies present on the five youngest apical leaves of each plant assessed.

The data recorded were used to calculate:

- The CMD incidence (%) using the following formula:

CMD Incidence (%) = (Number of infected plants/Total number of plants assessed) × 100;

- The severity of CMD symptoms was scored using a scale of 1–5, with 1 representing no symptom and 5 representing very severe symptoms. The mean severity (Sm) was calculated using the following formula:

Sm = Σ scores of diseased plants/Total number of diseased plants;

- The infection was categorized as either whitefly-borne or cutting-borne. Where CMD symptoms were present only on the upper leaves of a plant, with no symptoms on the lower leaves, the infection was considered to be whitefly-borne. Where only the lowest leaves and/or all leaves of a plant showed CMD symptoms, the infection was considered to be cutting-borne. The percentage of plants infected by cuttings or whiteflies was calculated based on infected plants only.

### 2.3. Cassava Variety Identification

The names of the cassava varieties growing in each field were documented during the survey. For well-known varieties, the surveyors recorded the names from a dropdown list on the digital field data collection tool. For varieties not recognized by the surveyors, the name of the cultivar was registered based on information provided by the farmers (where present). Otherwise, the cultivars were described according to the morphological descriptors compiled by Fukuda et al. [19] and Djaha et al. [20]. The descriptors include the color of the petiole, the color of the apical leaves and the color of the leaves. Each cultivar with distinctive characteristics was assigned a unique identification code.

### 2.4. Molecular Analysis

Total DNA was extracted from the cassava leaf samples collected following the cetyltrimethylammonium bromide (CTAB) protocol established by Doyle and Doyle [21]. The dried DNA pellet was resuspended in 100 µL RNase-free water, and DNA concentration and purity was measured using a NanoDrop OneC spectrophotometer (Thermo Fisher Scientific, Waltham, MA, USA).

Specific primer pairs designed for the detection of cassava mosaic begomovirus (CMBs) were used for the polymerase chain reaction (PCR) (Table 1). PCR mixtures (20 μL) were prepared using the 5× FIREPol Master Mix Ready to Load (Solis BioDyne, Tartu, Estonia), which includes FIREPol DNA polymerase, reaction buffer, 12.5 mM MgCl_2_, 1 mM dNTPs and loading dye. For each reaction, 0.3 µM of each primer was used. PCR reactions were carried out in the Eppendorf Thermocycler (Nexus gradient AG 22331 Hamburg, Germany) using the following PCR cycling conditions: denaturation at 94 °C for 4 min, followed by 35 cycles of denaturation at 94 °C for 1 min, annealing at 55 °C for 1 min and extension at 72 °C for 1 min. A final extension step was performed at 72 °C for 10 min. The PCR products were separated by electrophoresis on a 1% agarose gel in TAE (1×) buffer. After electrophoresis, the gel was stained with ethidium bromide and visualized under ultraviolet light (UV Transilluminator-26, VWR, Philadelphia, PA, USA).

### 2.5. Sequencing and Phylogenetic Analysis

To confirm the presence of EACMV-Ug in the samples, all samples that tested positive for the WAVE177F/WAVE569R and JSP001/JSP003 primers were systematically sequenced. Sequencing was performed by Genewiz (Germany, Leipzig) using the Sanger method. Raw sequence data were cleaned and assembled into contigs using Geneious Prime version 2025.0.3. The contigs were subjected to BLAST+ 2.17.0 searches against the NCBI database to identify homologous viral sequences. For phylogenetic analysis, representative begomovirus sequences known to infect cassava were retrieved from GenBank.

Sequences were aligned with representative isolates of CMBs using ClustalW in MEGA version 11. Phylogenetic and molecular evolutionary relationships were examined by analyzing partial viral sequences of the amplified virus using MEGA 11. Phylogenetic trees were constructed with the Maximum Likelihood (ML) method under the General Time Reversible (GTR) model, with 1000 bootstrap replicates to assess branch support.

### 2.6. Statistical Analysis

Epidemiological data were analyzed using R v. 4.5.1 [25]. In order to compare the incidence, mean severity of CMD, mode of infection and abundance of whitefly between the two border areas surveyed, Student’s *t*-tests for independent samples were performed. The Student’s *t*-test was conducted using field-level means. Plots and maps were generated using the ggplot2 package [26]. To study how virus species affect the severity of the symptoms observed, we determined the conditional probabilities of different events. The statistical significance of the different conditional probabilities was tested using binomial tests, and the results were used to determine 95% confidence intervals.

## 3. Results

### 3.1. Detection of CMBs in Samples from the 2022 Nationwide Survey in Côte d’Ivoire

In total, 357 fields were surveyed, and 737 cassava leaf samples were collected in 2022. Molecular analyses revealed the presence of African cassava mosaic virus (ACMV) and East African cassava mosaic Cameroon virus (EACMCMV). East African cassava mosaic virus-Uganda (EACMV-Ug) was not detected. The overall infection rate was 75.98%. Single ACMV infection and single EACMCMV infection were observed in 34.19% and 3.26% of the samples tested respectively. The double co-infection of ACMV and EACMCMV was predominant, with an infection rate of 38.53% (Table 2).

### 3.2. Detection of CMBs in Samples from the Focus Survey Conducted in Western Côte d’Ivoire in 2025

**Figure 1:**
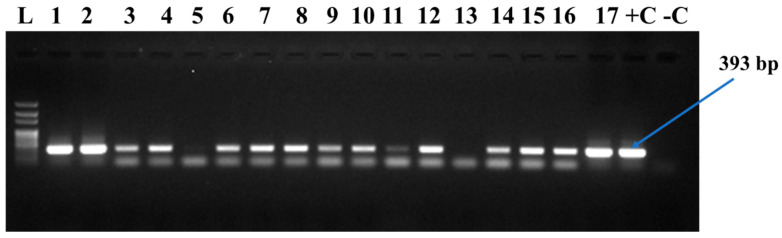
Electrophoresis gel (1% agarose) of PCR products for the detection of cassava mosaic begomoviruses. L = 100 bp DNA ladder, 1–17 = samples tested, +C = positive control, −C = negative control.

A total of 191 fields was surveyed across the western border of Côte d’Ivoire in 2025, with 95 fields located along the Liberian border and 96 fields located along the Guinean border. In total, we collected 551 cassava leaf samples, 305 along the Liberian border and 246 along the Guinean border. Molecular analyses of the 551 leaf samples collected confirmed the presence of three (3) cassava mosaic begomoviruses at the western border of Côte d’Ivoire, including ACMV, EACMCMV, and EACMV-Ug (Table 3 and Figure 1). The overall infection rate was 98.37% (542/551) and two types of single infections were detected, ACMV or EACMCMV. The rate of single ACMV infection was lower (3.09%;17/551), compared to single EACMCMV infection (15.79%; 87/551). Two types of double co-infection (co-infection with two viruses) were detected: ACMV+EACMCMV and EACMCMV+EACMV-Ug (Table 3). The ACMV+EACMCMV double co-infection rate was significantly higher, detected in 55.90% (308/551) of the samples tested, while the EACMCMV+EACMV-Ug combination was particularly rare, occurring only in 0.18% of the samples tested (1/551). Meanwhile, the rate of the triple co-infections (co-infection with three viruses) ACMV+EACMCMV+EACMV-Ug was relatively high (23.41%; 129/551). In this study, taking into account all samples tested (551), EACMV-Ug obtained an infection rate of 23.51% (130/551).

**Figure 2:**
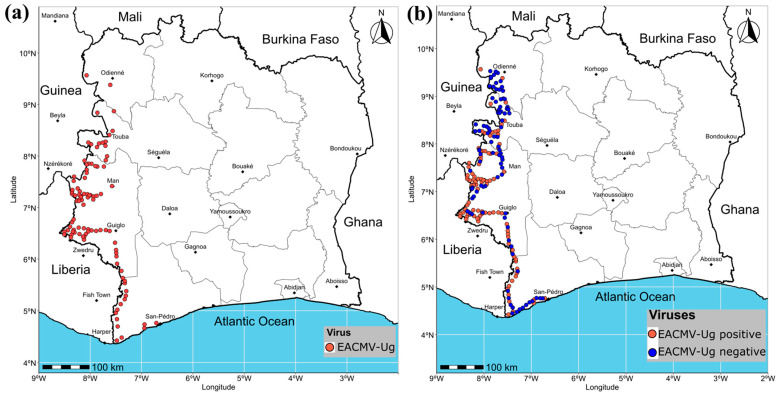
Distribution of cassava mosaic begomoviruses in cassava fields in western Côte d’Ivoire: (**a**) distribution of EACMV-Ug along the western borders of Côte d’Ivoire in 2025, (**b**) distribution of cassava fields with EACMV-Ug positive samples and fields without EACMV-Ug infection in western Côte d’Ivoire in 2025.

A comparison of the results from the two border zones revealed that both zones had very high infection rates. The Liberian side had an infection rate of 98.03% (299/305), while the Guinean border had a 98.78% (243/246) infection rate. In addition, the infection rate of the ACMV+EACMCMV+EACMV-Ug triple co-infections was significantly higher along the Liberian border (28.52%; 87/305), compared to 17.07% (42/246) along the Guinean border (Table 3). The distribution of EACMV-Ug along the western border of Côte d’Ivoire is shown in Figure 2a, while Figure 2b illustrates the distribution of cassava fields with EACMV-Ug positive samples and fields without EACMV-Ug infection in western Côte d’Ivoire. These results confirm the presence of EACMV-Ug in the Ivorian territory (Figure 2).

### 3.3. CMD Epidemiological Assessment in Western Côte d’Ivoire

**Figure 3:**
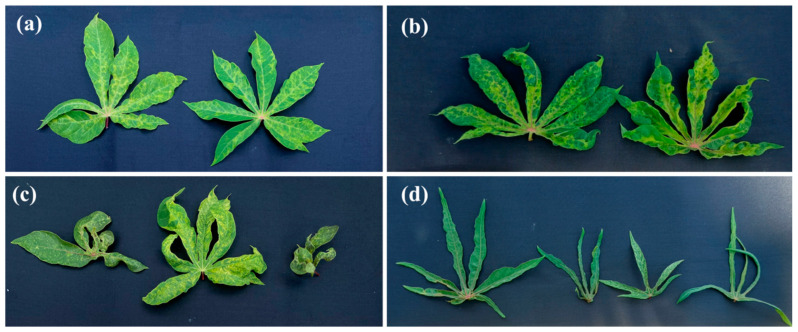
Symptoms of cassava mosaic disease (CMD) observed in cassava fields in western Côte d’Ivoire in 2025: (**a**) mild mosaic; (**b**) severe mosaic and slight leaf distortion; (**c**) mosaic, vein banding and leaf distortion; (**d**) filiform leaves.

**Figure 4:**
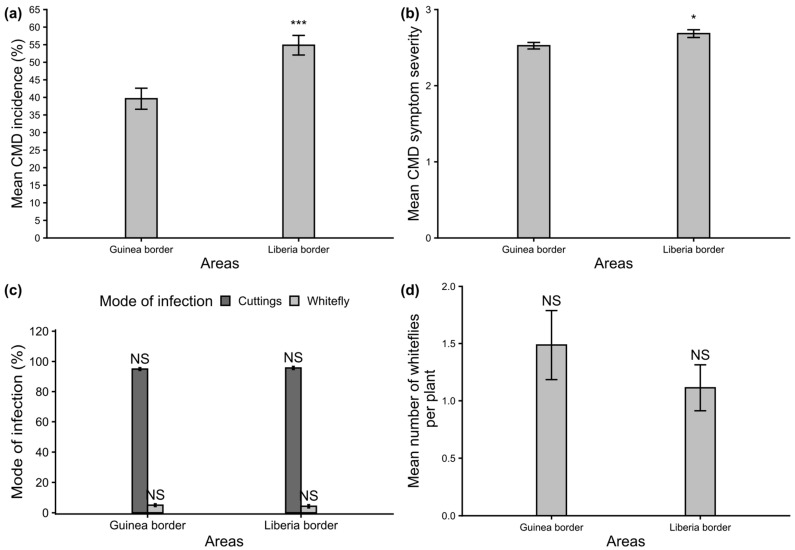
Epidemiological assessment of cassava mosaic disease (CMD) along the western border of Côte d’Ivoire: (**a**) mean CMD incidence, (**b**) mean CMD symptom severity, (**c**) mode of infection by cuttings and whiteflies; (**d**) mean number of whiteflies per plant. The error bars represent the standard error (SE). The bars marked *** indicate a significant difference at *p* < 0.001 between areas, * indicates a significant difference at *p* < 0.05, NS, not significant (α = 0.05).

**Figure 5:**
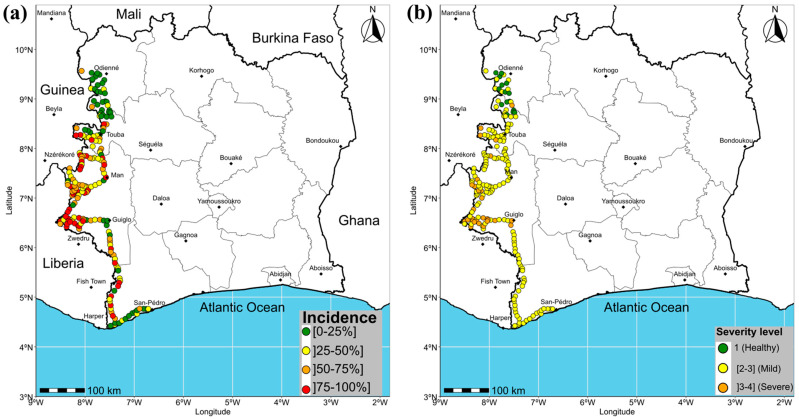
CMD epidemiological maps along the western border of Côte d’Ivoire in 2025 (**a**) mean CMD incidence per field (**b**) mean CMD severity per field.

Different types of CMD symptoms were observed in the cassava field in western Côte d’Ivoire. These symptoms included mosaic, leaf deformations, vein banding and filiform leaves (Figure 3). The overall CMD incidence was relatively moderate across the entire western border of Côte d’Ivoire, reaching an average of 47.19 ± 0.62%. The mean symptom severity was also moderate, with a value of 2.48 ± 0.25. The mean CMD incidence along the border with Liberia (54.84 ± 0.5%) was significantly higher (*p* < 0.001) than that on the Guinean border (39.62 ± 0.74%; Figure 4a). A significant difference (*p* < 0.05) was also observed between the mean symptom severity along the Liberian border (2.68 ± 0.19) and the mean symptom severity along the Guinean border (2.27 ± 0.30; Figure 4b). The mean incidence and mean symptom severity of individual fields surveyed is shown in Figure 5a,b.

### 3.4. Mode of Infection and Whitefly Abundance Along the Western Border of Côte d’Ivoire

The percentage of cutting-borne infection observed along the entire western border of Côte d’Ivoire was 95.35% ± 0.32% and 4.65% ± 2.09% for whitefly-transmitted infections. The use of infected cassava cuttings was therefore the main mode of CMD infection in the region. Additionally, the rate of cutting-borne infections was not significantly different between the Liberian border (95.72 ± 0.11%) and the Guinean border (94.98 ± 0.46%), as shown in Figure 4c. There was no significant difference between the two border areas for the whitefly infection rates (5.02 ± 1.8%) on the Guinean side and 4.28 ± 2.35% on the Liberian side (Figure 4c). The mean whiteflies abundance per plant was relatively low, with an average of 1.30 ± 0.31. However, the Guinean border area had a slightly higher average (1.49 ±0.30) than the Liberian border (1.11 ± 0.20; Figure 4d).

### 3.5. Relationship Between the Types of Infection and CMD Symptom Severity

The samples obtained during the survey were categorized into four unique groups according to their phytosanitary status and symptom severity. Based on this categorization, there were 201 asymptomatic samples, 173 with mild symptoms, 105 with severe symptoms, and 58 exhibiting very severe symptoms. Binomial tests on the established conditional probabilities indicated that the likelihood of identifying a single ACMV infection in asymptomatic, mild, severe, and very severe samples was comparatively low, with values varying between 1.72% and 3.81% (Table 4). Regarding single EACMCMV infections, a higher value (34.83%) was observed in asymptomatic samples. However, this single infection was significantly low in samples with mild (5.20%), severe (4.76%), or very severe (5.17%) symptoms. With regard to double co-infection, the EACMCMV+EACMV-Ug combination was rarely found (0.58%) whereas the ACMV+EACMCMV double co-infection was predominant, detected in 60.70% of asymptomatic samples, 57.80% of samples with mild symptoms, 51.43% of severe samples, and 55.17% of very severe samples (Table 4). The triple co-infection ACMV+EACMCMV+EACMV-Ug was rarely detected in asymptomatic samples (1.49%). However, the probability of encountering this viral combination is higher in samples displaying severe (40%) and very severe (37.93%) CMD symptoms compared to samples with mild samples (32.95%) (Table 4).

### 3.6. Relationship Between Cassava Varieties and Infecting Begomoviruses

**Figure 6:**
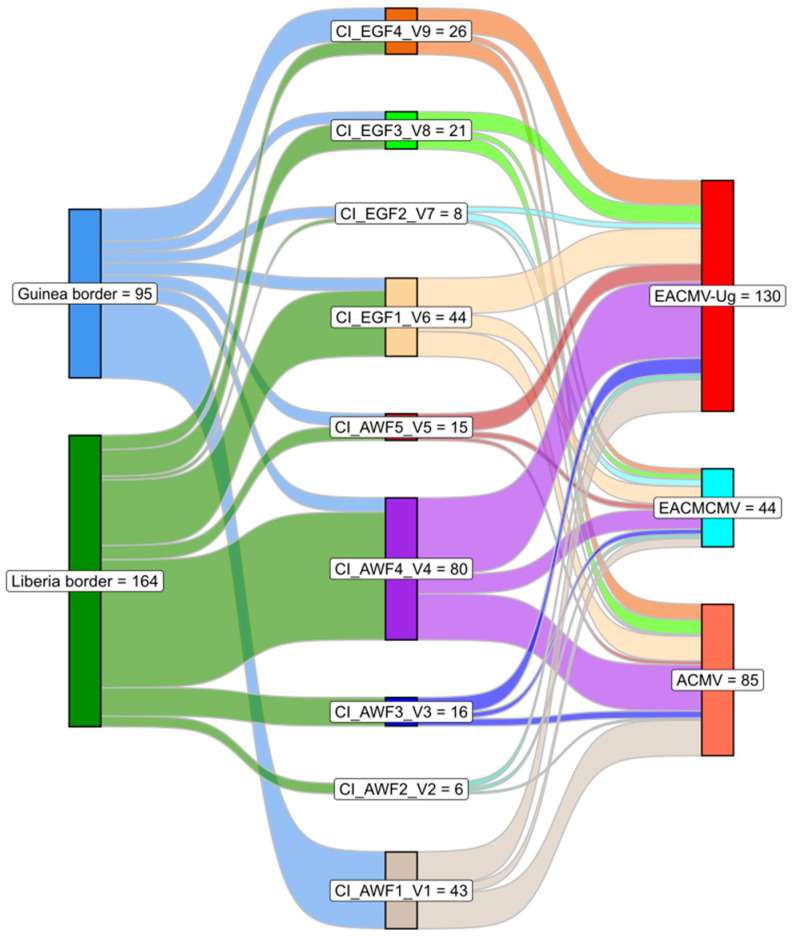
Sankey diagram illustrating the affinity of nine cassava varieties identified along the Western borders of Côte d’Ivoire in 2025 to three cassava mosaic begomoviruses. The width of each node and the number of flow lines are proportional to the number of infected samples of the same cassava variety.

Most farmers were absent from their fields during the survey. Based on the morphological characteristics the following codes were assigned to the nine uniquely distinguishable varieties as follows: CI_AWF1_V1, CI_AWF2_V2, CI_AWF3_V3, CI_AWF4_V4, CI_AWF1_V5, CI_EGF1_V6, CI_EGF2_V7, CI_EGF3_V8, and CI_EGF4_V9 (Table 5). All the identified cassava varieties found on the western border of Côte d’Ivoire were infected by at least one begomovirus. Figure 6 illustrates the relationship between cassava varieties and infecting cassava begomoviruses. All nine varieties could be infected by ACMV, EACMCMV, and EACMV-Ug. EACMV-Ug showed a significant infection rate in the CI_AWF4_V4 (33.07%), CI_EGF1_V6 (15.38%), and CI_AWF1_V1 (13.84%) varieties. Variety CI_AWF4_V4, which showed the highest susceptibility to EACMV-Ug, was mainly found along the border with Liberia. CI_AWF2-V2 and CI_EGF2_V7 appeared to be the least affected by EACMV-Ug, with an infection rate of only 2.30% each (Figure 6). CI_AWF2_V2 was only found along the border with Liberia, while CI_EGF2_V7 was grown along the entire western border. As for EACMCMV, its infection rate was particularly high in CI_AWF4_V4 (25%) and CI_EGF1_V6 (22.72%). In contrast, CI_AWF2_V2 had the lowest EACMCMV infection rate (4.54%). The EACMCMV infection rate for the other varieties ranged from 6.81% to 11.36%. CI_AWF4_V4 (30.58%), CI_AWF1_V1 (23.53%), and CI_EGF1_V6 (16.47%) were the most susceptible varieties to ACMV infection, whereas CI_EGF2-V7 (1.17%) and CI_AWF5_V5 (2.35%) were the least affected (Figure 6).

### 3.7. Phylogenetic Relationship Between EACMV-Ug Coat Protein Genes

**Figure 7:**
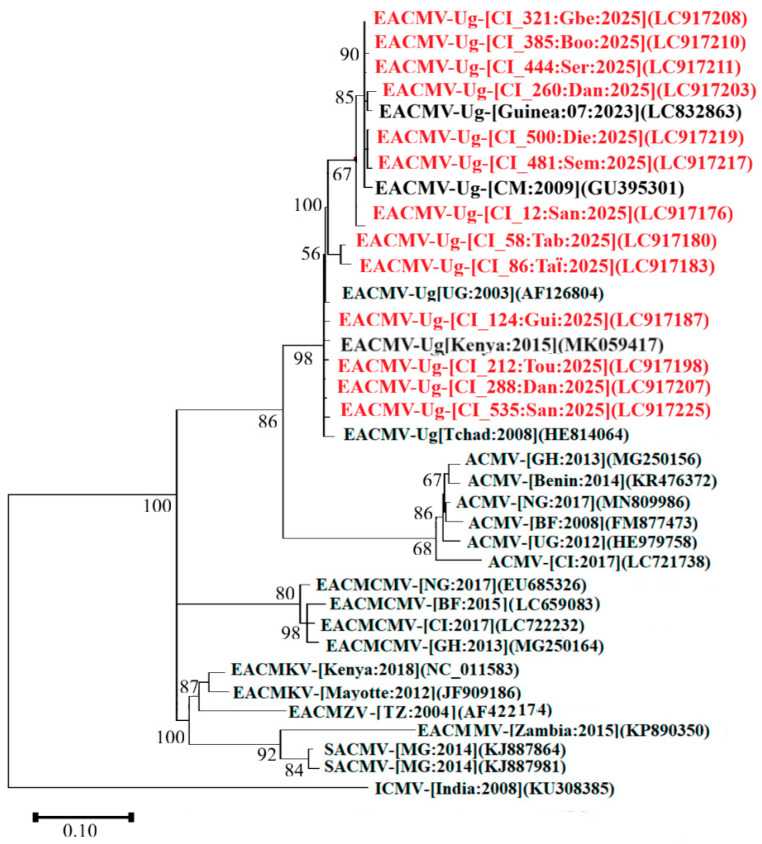
Maximum likelihood phylogenetic tree showing the relationships between Côte d’Ivoire isolates of East African cassava mosaic-Uganda virus (EACMV-Ug; 13 isolates) and diverse isolates representative of cassava mosaic begomovirus diversity. The tree is based on partial sequences for EACMV-Ug (DNA-A coat protein) and rooted using Indian cassava mosaic virus (GenBank accession number, DNA-A: KU308385) as an outgroup. Sequences obtained in this study are colored in red while those in black were obtained from GenBank. Bootstrap analysis was performed with 1000 replicates.

For the identification of EACMV-Ug in samples, a total of 266 samples were sequenced using the WAVE177F/WAVE569R and JSP001/JSP003 primers cited in Table 1. Analysis of the sequences obtained revealed that 48.87% (130/266) of the resulting sequences had high similarity to the EACMV-Ug variant, with nucleotide identity ranging from 97.20% to 99.21%. A total of 50 sequences was submitted to GenBank under accession numbers LC917176-LC9117225. Among these, 13 representative sequences, i.e., well distributed across the entire western border, were used to construct the phylogenetic tree. Phylogenetic analysis performed using these sequences and sequences retrieved from the GenBank clearly confirmed that the sequences obtained in this study belong to the EACMV-Ug group (Figure 7). Furthermore, the phylogenetic tree showed that these sequences are closely related to EACMV-Ug sequences from Guinea (LC832863), Cameroon (GU395301), Chad (HE814064), Uganda (AF126804), and Kenya (AJ711530, MK059417, JN053446) (Figure 7). These results definitively confirm the presence of EACMV-Ug in Côte d’Ivoire.

## 4. Discussion

Cassava is a vital crop in Central and West Africa (CWA) and is essential for food security in the region. To ensure sustainable cassava production, the Central and West African Virus Epidemiology (WAVE) program (https://wave-center.org/our-identity/, accessed on 30 Ocotber 2025) has conducted epidemiological surveys in 14 CWA countries towards a regional coordinated effort to manage the viruses that constitute a major constraint to cassava production. As a result of these surveys, Combala et al. [15] reported for the first time the presence of the EACMV-Ug variant in Guinea and Sierra Leone and raised the alert on a possible eastward front of CMD developing in West Africa. Since EACMV-Ug was not detected in any of the samples collected during the 2022 cassava field surveys in Côte d’Ivoire and given the country’s proximity to Guinea, we conducted another survey in 2025, focusing on the western Ivorian border, to generate up-to-date epidemiological data to inform evidence-based decision making for preparedness planning.

Molecular analysis of cassava leaf samples collected during the nationwide cassava field survey conducted in Côte d’Ivoire in 2022 did not reveal the presence of EACMV-Ug in the country at that time. Although the 2022 survey did not specifically target the western border of the country, several sites in the area were still visited. Therefore, the major finding of this study, the identification of the EACMV-Ug variant in Côte d’Ivoire for the first time in 2025, suggests a relatively recent incursion of this variant in the country and its establishment and relatively rapid spread through the use of infected planting materials. Indeed, the molecular analysis conducted showed a high infection rate of EACMV-Ug in the western border areas.

The CMD epidemic which devastated cassava fields in Uganda in the 1990s was a classic textbook virus epidemic. This epidemic was caused by a combination of biological factors: the recombination between ACMV and EACMV that resulted in the more virulent EACMV-Ug, the high affinity between EACMV-Ug and the whitefly vector *Bemisia tabaci*, an increase in whitefly populations at the front of the epidemic, the synergistic interaction between EACMV-Ug and ACMV and the widespread cultivation of a susceptible cassava variety named Ebwanatereka [23]. The lessons learnt from the Ugandan CMD epidemic prompted us to investigate the role of these various factors in the rapid establishment of EACMV-Ug along the western border of Côte d’Ivoire.

The whitefly vector, *Bemisia tabaci*, was one of the main factors which contributed to the rapid spread of EACMV-Ug in Uganda since the CMD epidemic was fueled by unusually high whitefly populations [27,28,29] of more than 100 whiteflies per plant [30,31]. Fortunately, in the case of the western Ivorian border, the whiteflies population in the fields was low, with an average of 1.3 ± 0.31 whiteflies per plant. This result suggests that *Bemisia tabaci* may not play a major role in the spread of EACMV-Ug in the western areas of Côte d’Ivoire. A similar low whitefly population trend was observed in Guinea (5.43 whiteflies per plant), and Sierra Leone (3.65 whiteflies per plant) [15,16]. However, the role of the whitefly in the spread of CMD in West Africa requires active monitoring. Indeed, previous WAVE studies have shown that the predominant whitefly species in the west of Côte d’Ivoire is SSA1-SG3 [32,33], a species which is highly efficient in the transmission of EACMV-Ug to cassava plants [34].

Apart from whitefly transmission, the use of infected cuttings is another important means for CMD propagation. This study reveals the infection rate of cutting-borne CMD infection in the western border of Côte d’Ivoire and suggests the continuous exchange of CMD-infected planting material between farmers in the area. Therefore, as a containment measure, we informed the Ivorian authorities and the major stakeholders in the cassava value chain of this finding and encouraged the enforcement of the use of certified disease-free planting material and a temporary ban on the movement of cassava cuttings from the western border areas of the country towards the mainland until a buffer zone is defined. In addition, efforts must be deployed to facilitate access to CMD-resistant varieties to stop this eastward spread of EACMV-Ug.

Most of the samples collected from the cassava varieties grown in the surveyed areas tested positive to at least one begomovirus, including asymptomatic samples. This suggests that the cassava varieties cultivated in the western part of Côte d’Ivoire are susceptible to CMBs. These varieties seem to be local ones, as reported by Djaha et al. [20]. Moreover, Amoakon et al. [35] showed that local varieties grown in Côte d’Ivoire are more susceptible to CMD compared to improved ones. Of the nine varieties identified during this survey, CI_AWF4_V4 appears to be the most susceptible to infection by EACMV-Ug. This variety was predominantly distributed along the border with Liberia, where EACMV-Ug was also found to be more widespread. In contrast, the less commonly cultivated varieties CI_AWF2_V2 and CI_EGF2_V7 were the only ones that showed reduced susceptibility to infection by CMBs.

As mentioned above, a key factor that led to the CMD epidemic in Uganda was the synergistic interaction between EACMV-Ug and ACMV, which resulted in more severe CMD symptoms. In a three-year study conducted in Côte d’Ivoire, Kouakou et al. [36] demonstrated a regression in the infection rate of single ACMV infection, which were gradually replaced by single EACMCMV infection in cassava fields in the country. Among the three viruses identified in this study, the percentage of single ACMV infection was found to be the lowest. This result is in contrast to a series of other investigations undertaken in West Africa [14,18] where ACMV was shown to be the main begomovirus infecting cassava. Furthermore, during the 2025 survey, EACMV-Ug was detected in co-infection with one or both of the other viruses already present in Côte d’Ivoire (ACMV and EACMCMV). The ACMV and EACMCMV combination was observed in significantly higher proportions (55.90%) compared to the EACMCMV and EACMV-Ug combination, which occurred very rarely (0.18%). Indeed, according to Pita et al. [23], there is a synergistic interaction between ACMV and EACMCMV when these viruses co-infect the same plant. However, the nature of the interaction between EACMCMV and EACMV-Ug under a co-infection condition is unknown and needs to be urgently investigated. The very rare occurrence of this double co-infection may suggest the possibility of an antagonistic interaction between EACMCMV and EACMV-Ug when they simultaneously infect the same plant. Indeed, antagonism between two begomoviruses is characterized by negative interactions during co-infection of the same host plant, leading to a decrease in the replication, accumulation, or transmission of one or both viruses compared to a single infection. Several studies have already demonstrated this antagonism between different begomoviruses, including *Orthotospovirus tomatomaculae* (Tomato spotted wilt virus; TSWV), *Begomovirus coheni* (Tomato yellow leaf curl virus; TYLCV), and *Begomovirus solanumvariati* (Tomato mottle virus; ToMoV) [37,38]. In contrast to the association between ACMV and EACMV-Ug, which was one of the key drivers of the Ugandan epidemic, we observed the predominance of EACMV-Ug in triple co-infection (ACMV+EACMCMV+EACMV-Ug) in the western border of Côte d’Ivoire. However, in this study, the triple infection was not necessarily associated with worsening symptoms, as the triple co-infection was observed in asymptomatic, moderate, severe, and very severe samples. Thus, it is clear that some of the trends observed in this study are not similar to those observed in Uganda in 1990. Still, particular attention should be given to these frequent cases of double and triple infections, which could lead to the emergence of an even more pathogenic variant.

The 2025 survey of the Western Ivorian border was prompted by the alert raised by Combala et al. [15] on the presence of EACMV-Ug in Guinea. Our data shows a higher infection rate of EACMV-Ug in the south-western part of the country bordering Liberia, compared to the north-western part bordering Guinea. This suggests with high probability the presence of EACMV-Ug in Liberia, where a nationwide cassava field survey must be conducted imperatively to protect cassava production in the country and ascertain the true status of the EACMV-Ug epidemic in West Africa.

## 5. Conclusions

This study highlights a significant risk to cassava production in Côte d’Ivoire by confirming the detection of EACMV-Ug in the country. Along with EACMV-Ug, ACMV and EACMCMV were also detected. Molecular analysis revealed single infections (ACMV and EACMCMV), double co-infections (ACMV+EACMCMV, EACMCMV+EACMV-Ug) and triple co-infections (ACMV+ EACMCMV+EACMV-Ug). EACMV-Ug was mainly detected in triple co-infection with ACMV and EACMCMV. The presence of EACMV-Ug in Côte d’Ivoire poses a real threat to cassava production in the country and in West Africa in general since it confirms the continuous transboundary eastward spread of the virus. The double and triple co-infections observed could lead to the emergence of a more virulent virus through recombination. Implementing contingency measures and deploying effective strategies such as limiting the movement of planting material between countries or between zones of the same country, and/or introduction of resistant varieties, enforcing the use of disease-free planting material is critical to limiting further spread of EACMV-Ug in Côte d’Ivoire and within West Africa. In addition, we recommend that country-wide cassava field surveys be conducted urgently to accurately assess the current extent of the spread of EACMV-Ug throughout Côte d’Ivoire and to investigate the probable presence of EACMV-Ug in Liberia.

## Figures and Tables

**Table 1:** List of primers used for PCR detection of cassava mosaic begomoviruses.

Primer Name	Primer Sequences (5′-3′)	Size (bp)	Virus Detected (Target Region)	References
WAVE177F	TCGAAGCCCAGATGTCCCTA	393	ACMV/EACMCMV/EACMV/ EACMV-Ug/AV1 (CP)	[15]
WAVE569R	CCACCAACAACAGTGGCATG			
WAVE-AA508F	AAGGCCCATGTAAGGTCCAG	800	ACMV/AV1/AC3	[22]
WAVE-AA1307R	GAAGGAGCTGGGGATTCACA			
JSP001	ATGTCGAAGCGACCAGGAGAT	780	EACMV/AV1 (CP)	[23]
JSP003	CCTTTATTAATTTGTCACTGC			
VNF031	GGATACAGATAGGGTTCCCAC	~560	EACMCMV/AC2/AC3	[24]
VNF032	GACGAGGACAAGAATTCCAAT			

**Table 2:** Percentage of cassava mosaic begomovirus detected in samples collected in Côte d’Ivoire in 2022.

Virus Detected						
Total	Negative Sample	EACMV-Ug	ACMV	EACMCMV	ACMV+EACMCMV	Infected Sample
737 (100%)	177 (24.02%)	00 (0.0%)	252 (34.19%)	24 (3.26%)	284 (38.53%)	560 (75.98%)

**Table 3:** Percentage of type of cassava mosaic begomovirus infections detected on the western border of Côte d’Ivoire in 2025.

Viruses Detected	Liberia Border	Guinea Border	Total
ACMV	10 (3.28%)	7 (2.85%)	17 (3.09%)
EACMCMV	51 (16.72%)	36 (14.63%)	87 (15.79%)
ACMV+EACMCMV	150 (49.18%)	158 (64.23%)	308 (55.90%)
EACMCMV+EACMV-Ug	1 (0.33%)	0 (0.00%)	1 (0.18%)
ACMV+EACMCMV+EACMV-Ug	87 (28.52%)	42 (17.07%)	129 (23.41%)
Total infected samples	299 (98.03%)	243 (98.78%)	542 (98.37%)
Negative samples	6 (1.97%)	3 (1.22%)	9 (1.63%)
Total	305 (100%)	246 (100%)	551 (100%)

**Table 4:** Relationship between type of infection and CMD symptom severity.

Symptoms Severity							
Type of Infection	Begomovirus(es)	Total	Asymptomatic	Mild	Severe	Very Severe	Confidence Interval
Single infection	ACMV	17	06	06	04	01	-
	Probability	-	(2.99%)	(3.47%)	(3.81%)	(1.72%)	[4 × 10^−4^, 9.4 × 10^−2^]
	EACMCMV	87	70	9	5	3	-
	Probability	-	(34.83%)	(5.20%)	(4.76%)	(5.17%)	[1 × 10^−4^, 4 × 10^−1^]
Double co-infection	ACMV+EACMCMV	308	122	100	54	32	-
	Probability	-	(60.70%)	(57.80%)	(51.43%)	(55.17%)	[4 × 10^−1^, 6 × 10^−1^]
	EACMCMV+EACMV-Ug	01	00	01	00	00	-
	Probability	-	-	(0.58%)	-	-	[0.0, 6 × 10^−2^]
Triple co-infection	ACMV+EACMCMV+EACMV-Ug	129	03	57	42	22	-
	Probability	-	(1.49%)	(32.95%)	(40.00%)	(37.93%)	[3 × 10^−3^, 5 × 10^−1^]
Total	-	542	201	173	105	58	-

**Table 5:** Morphological characteristics of the 9 cassava varieties.

Cassava Varieties	Color of Apical Leaves	Petiole Color	Leaf Color
CI_AWF1_V1	Dark green	Yellowish-green	Light green
CI_AWF2_V2	Purple	Purple	Purple green
CI_AWF3_V3	Purplish green	Green	Dark green
CI_AWF4_V4	Light green	Red	Dark green
CI_AWF1_V5	Purple	Red	Purple green
CI_EGF1_V6	Dark green	Greenish-red	Dark green
CI_EGF2_V7	Light green	Greenish-red	Purple green
CI_EGF3_V8	Dark green	Red	Light green
CI_EGF4_V9	Purplish green	Green	Light green

## Data Availability

The dataset supporting the findings, and the results of this study are provided in the manuscript.
